# Avian Influenza among Waterfowl Hunters and Wildlife Professionals

**DOI:** 10.3201/eid1208.060492

**Published:** 2006-08

**Authors:** James S. Gill, Richard Webby, Mary J.R. Gilchrist, Gregory C. Gray

**Affiliations:** * University of Iowa Hygienic Laboratory, Iowa City, Iowa, USA;; † St Jude Children's Research Hospital, Memphis, Tennessee, USA;; ‡ University of Iowa College of Public Health, Iowa City, Iowa, USA

**Keywords:** avian influenza, hemagglutinin 11, waterfowl hunters, wildlife professionals, microneutralization, hemagglutination inhibition, dispatch

## Abstract

We report serologic evidence of avian influenza infection in 1 duck hunter and 2 wildlife professionals with extensive histories of wild waterfowl and game bird exposure. Two laboratory methods showed evidence of past infection with influenza A/H11N9, a less common virus strain in wild ducks, in these 3 persons.

Wild ducks, geese, and shorebirds are the natural reservoir for influenza A virus (*1*); all 16 hemagglutinin (H) and 9 neuraminidase (N) subtypes are found in these wild birds (*1**,**2*). Recently, the rapid spread of influenza A/H5N1 virus to new geographic regions, possibly by migrating waterfowl, has caused concern among public health officials who fear an influenza pandemic. Until now, serologic studies of the transmission of subtype H5N1 and other highly pathogenic strains of avian influenza have focused on humans who have contact with infected domestic poultry (*3**,**4*). In this cross-sectional seroprevalence study, we provide evidence of past influenza A/H11 infection in persons who were routinely, heavily exposed to wild ducks and geese through recreational activities (duck hunting) or through their employment (bird banding). To our knowledge, this study is the first to show direct transmission of influenza A viruses from wild birds to humans.

## The Study

In mid-October 2004, we enrolled 39 duck hunters who were hunting in southeastern Iowa at Lake Odessa Wildlife Management Area, the state's only limited-access public waterfowl hunting area managed by the Iowa Department of Natural Resources (DNR). In February 2005 we enrolled 68 Iowa DNR employees, many of whom had duck hunted or had been involved annually in capturing and banding wild ducks and geese as part of their duties of employment. Ten (15%) of the 68 DNR workers reported no contact with ducks. The duck-hunting group consisted of men >16 years of age, and the DNR group consisted of 65 men and 3 women enrollees. The average age of the duck hunters and DNR workers was 34 and 47 years, respectively. The average number of years of waterfowl or bird exposure of the duck hunters and DNR workers was 19.8 and 21.5, respectively. In the 3 years before the study, influenza vaccine had been administered to 37% of the duck hunters and 35% of the DNR workers.

Microneutralization assay, adapted per Rowe et al. (*5*), was performed on all serum samples with influenza A subtypes H1 through H12 from avian sources. Virus at 100 TCID_50_ (50% tissue culture infective dose)/50 μL was incubated at 37°C for 2 h with heat-inactivated serum in 96-well plates. One hundred microliters of trypsinized London MDCK cells at 2 × 10^5^ cells/mL, grown to 70%–95% confluency, was added to each well. After 24 h at 37°C, the cells were acetone-fixed, and horseradish peroxidase–based ELISA was performed with mouse-specific anti-influenza A antibody. Optical density was read at 450 nm. All tested virus isolates were titrated with and without trypsin in the University of Iowa's Emerging Pathogens Laboratory; no significant difference in titers was observed. Backtiter controls were performed with each microneutralization assay.

Hemagglutination inhibition (HI) assay with horse erythrocytes, adapted per Meijer et al. (*6*), was performed on all hunter serum samples by using avian influenza A subtype H11. Heat-inactivated serum treated with receptor-destroying enzyme was first heme-adsorbed with packed horse erythrocytes. Serum was then incubated with virus at 8 hemagglutinin U/50 μL with 1% horse erythrocytes in 0.5% bovine serum albumin in phosphate-buffered saline for 1 h at room temperature in V-bottom plates. The plates were then examined.

One 39-year-old duck hunter had a titer of 40, and 2 male DNR workers, ages 52 and 53, had titers of 10 against influenza A/H11N9/duck/Memphis/546/76 by microneutralization assay (Table). These 3 study participants had substantial lifetime exposures to wild waterfowl. The duck hunter and the 2 DNR workers had 31, 27, and 30 years of duck-hunting experience, respectively. The duck hunter spent 25–60 days in the marsh each year hunting ducks. He harvested 100 ducks annually and handled another 300 ducks with his hunting partners during the duck-hunting season from mid-September to early December. One of the positive DNR workers (age 52) had several years of live wild duck–banding exposure as part of his annual duties of employment, in addition to 27 years of duck-hunting exposure. Each year this wildlife professional had contact with >100 live ducks during the banding season in late August and early September. Serum samples from all other study participants were negative against subtype H11N9 according to results of microneutralization assay and horse erythrocyte HI assays. The duck hunter's serum was not reactive to any other avian influenza hemagglutinin subtypes tested (H1–H10 and H12). The sera of the 2 H11-positive DNR workers had titers of 10 for influenza A/H2N2/mallard/NY/6750/78 according to microneutralization assay results and were negative for H1, H3–H10, and H12. Results of the H11 microneutralization assay were verified by horse erythrocyte HI assay that used subtype H11N9 virus. The titers by horse HI assay of the microneutralization assay–positive duck hunter and the 2 DNR workers were 10 or 20 (Table). These 3 study participants had not been vaccinated against influenza within 3 years before the study.

**Table:** Serologic results and demographics of duck hunter and Iowa DNR workers*

Waterfowl handlers	Sex	Age, y	Hunting/bird exposure, y	MN titer	HI titer
Hunter	M	39	31	40	10
DNR 1	M	52	27	10	10†
DNR 2	M	53	30	10	10

*DNR, Department of Natural Resources; MN, microneutralization assay; HI, hemagglutination inhibition. †Repeat HI titer was 20.

## Conclusions

Virus transmission from wild waterfowl to humans has not been documented. To our knowledge this study is the first to assess hunters with substantial exposures to wild ducks and geese, the known natural reservoir of influenza A virus in nature (*1*). During late August and early September in Iowa, when the banding of wild ducks occurs, and in mid-September, when duck hunting begins, a significant proportion of hatch-year mallards (up to 65%) and other ducks may be infected with influenza A virus according to other studies in North America (*1**,**7*). Later in the season, as the duck migration progresses, a decrease in prevalence is commonly seen (*1**,**8*). In late August 2004, we isolated influenza virus from mallards (60%) and from wood ducks (13%) in Iowa (data not shown).

Even though the H11-positive study participants had several years of exposure to wild birds infected with avian influenza virus through hunting and duck banding, they did not wear personal protective equipment, such as gloves, masks, or eye protection. These participants also did not use tobacco, a recently identified risk factor among swine facility workers with elevated serum antibodies against swine strains of influenza (*9*).

In this study we did not attempt to associate disease symptoms with exposure to wild waterfowl. Others have shown that domestic bird–acquired influenza A/H7N7 in humans may frequently lead to minor illness, such as conjunctivitis (*4**,**10**,**11*), although more serious disease has been recorded (*4**,**10*). We provide serologic evidence from 2 assays, microneutralization assay and horse erythrocyte HI, for past infection in humans with avian influenza A/H11 and no other avian influenza subtypes. Our findings are consistent with those of Beare and Webster (*12*), who reported a lack of antibody response in human volunteers inoculated with avian influenza strains with HA antigens wholly alien to humans. Those researchers did not inoculate volunteers with H11. In our study, a less common hemagglutinin subtype (H11) has apparently caused serologically detectable infections in high-exposure groups, whereas the more common hemagglutinin subtypes H4 and H6 (*13**–**15*) in wild ducks have not. The reason for this finding is unknown but may include the following: 1) H11 may have increased ability to infect humans, 2) H11 may provoke a relatively strong and detectable immune response, and 3) our serologic assays may be more sensitive in detecting H11 infection than other H subtypes.

Even though none of the H11-positive study participants had received influenza vaccine within the previous 3 years, the 2 positive DNR workers also showed reactivity by microneutralization assay to avian subtype H2N2. This result was not unexpected and likely represents reactivity from natural infection of the human H2N2 strain derived from avian sources that circulated from 1957 to 1967. Forty-one percent of participants of similar age (range 43–68 years, average 56 years) who grew up during the era of the human H2N2 pandemic also had positive test results. Except for the 2 H11N9-positive DNR workers, the other H2N2-positive study participants were nonreactive against avian subtype H11N9 (data not shown). This finding strengthens our conclusion that there was no cross-reactivity between H2N2 and H11N9 antisera. None of H11-positive study participants was reactive to avian subtypes H1 or H3, although others in the study population were. Only 7% and 18% of the study population were reactive by microneutralization assay against H1 and H3, respectively.

The relative lack of antibody response in our study population, who had substantial exposures to waterfowl with influenza A infections, and in inoculated volunteers from Beare and Webster (*12*) suggests that avian influenza infections in humans exposed to wild waterfowl may occur more commonly than we are able to detect with current methods. Although the sample size of our study was relatively small, our results suggest that handling wild waterfowl, especially ducks, is a risk factor for direct transmission of avian influenza virus to humans.
